# Draft Whole-Genome Sequence of Bacillus altitudinis Strain B-388, a Producer of Extracellular RNase

**DOI:** 10.1128/genomeA.01502-14

**Published:** 2015-01-29

**Authors:** Raihan Shah Mahmud, Vera Ulyanova, Sergey Malanin, Elena Dudkina, Valentina Vershinina, Olga Ilinskaya

**Affiliations:** Institute of Fundamental Medicine and Biology, Kazan (Volga Region) Federal University, Kazan, Russia

## Abstract

Here, we present a draft genome sequence of Bacillus altitudinis strain B-388, including a putative plasmid. The strain was isolated from the intestine of Indian meal moth, a common pest of stored grains, and it is characterized by the production of extracellular RNase, similar to binase, which is of interest for comparative studies and biotechnology.

## GENOME ANNOUNCEMENT

The species Bacillus altitudinis was described for the first time in 2006 as a microorganism found in air samples collected at an altitude of 41 km (1). Later, it was isolated from slaughterhouse soil (2), the digestive tract of a fish (3), silt (4), and plant rhizosphere (5). B. altitudinis strain B-388 was initially secured as Bacillus entomocidus from the intestinal tract of Plodia interpunctella (Indian meal moth), a common pest of stored grains, in the 1950s; it was recently reidentified using molecular methods. It produces an extracellular guanyl-preferring RNase similar to binase (6), an RNase secreted by a lately sequenced strain of Bacillus pumilus (7) and possessing antitumor and antiviral properties (8, 9). It also can be used as an RNA-degrading tool. The whole-genome shotgun sequencing project reported here is the second one for the species, following that of its type strain, B. altitudinis 41KF2b (DDBJ/EMBL/GenBank accession no. ASJC00000000.1).

The sequencing of B. altitudinis B-388 genomic DNA was performed on a 454-GS Junior system (Roche, USA), with approximately 20-fold overall genome coverage. The obtained 157,508 reads were assembled with the GS *de novo* Assembler (Newbler software suite, version May 2014), resulting in 59 contigs (>200 bp) with an *N*_50_ size of 127,734 bp. The calculated genome size was 3,706,590 bp, with a G+C content of 41.2 mol%. Contig 45 represents a putative plasmid of 4,528 bp. The genome was annotated using the NCBI Prokaryotic Genome Annotation Pipeline (PGAP) version 2.6 (http://www.ncbi.nlm.nih.gov/genomes/static/Pipeline.html). A total of 3,730 genes and 3,555 coding sequences (CDSs) were predicted, and 77 RNAs were identified, including 71 tRNAs, 5 rRNAs, and 1 noncoding RNA (ncRNA). The total size of the assembly, G+C content, and the numbers of coding sequences and RNAs are in agreement with the respective figures for the type strain of B. altitudinis 41KF2b (3,678,935 bp, 41.3 mol%, 3,800 CDSs, and 55 RNAs).

A subsequent analysis of the genome content of B. altitudinis B-388 will help to determine the key aspects of its interaction with the environment and conduct thorough comparative studies with the closest phylogenetic neighbor B. pumilus, which produces extracellular RNase bearing only 1 amino acid substitution compared with the B. altitudinis RNase.

### Nucleotide sequence accession number.

This whole-genome shotgun project has been deposited in DDBJ/EMBL/GenBank under the accession no. JOVS00000000. The version described in this paper is the first version.
